# Multimedia Fusion Privacy Protection Algorithm Based on IoT Data Security under Network Regulations

**DOI:** 10.1155/2022/3574812

**Published:** 2022-08-31

**Authors:** Guicun Zhu, Xingguo Li, Changlong Zheng, Linlin Wang

**Affiliations:** ^1^Department of Economic Management, Dongchang College of Liaocheng University, Liaocheng, Shandong 252000, China; ^2^Dongchang Middle School of Liaocheng Economic and Technological Development Zone, Liaocheng, Shandong 252000, China; ^3^Liaocheng Yucai School, Liaocheng, Shandong 252000, China

## Abstract

This study provides an in-depth analysis and research on multimedia fusion privacy protection algorithms based on IoT data security in a network regulation environment. Aiming at the problem of collusion and conspiracy to deceive users in the process of outsourced computing and outsourced verification, a safe, reliable, and collusion-resistant scheme based on blockchain is studied for IoT outsourced data computing and public verification, with the help of distributed storage methods, where smart devices encrypt the collected data and upload them to the DHT for storage along with the results of this data given by the cloud server. After testing, the constructed model has a privacy-preserving budget value of 0.6 and the smallest information leakage ratio of multimedia fusion data based on IoT data security when the decision tree depth is 6. After using this model under this condition, the maximum value of the information leakage ratio of multimedia fusion data based on IoT data security is reduced from 0.0865 to 0.003, and the data security is significantly improved. In the consensus verification process, to reduce the consensus time and ensure the operating efficiency of the system, a consensus node selection algorithm is proposed, thereby reducing the time complexity of the consensus. Based on the smart grid application scenario, the security and performance of the proposed model are analyzed. This study proves the correctness of this scheme by using BAN logic and proves the security of this scheme under the stochastic prediction machine model. Finally, this study compares the security aspects and performance aspects of the scheme with some existing similar schemes and shows that the scheme is feasible under IoT.

## 1. Introduction

With the rapid development of new-generation information technology such as cloud computing and edge computing, the global data volume is growing massively. Data have become an important strategic resource that affects global competition, and countries have introduced corresponding policies to support the development of the digital economy, aiming to promote the full utilization of data value in various industries [1]. However, data security incidents have occurred frequently in recent years, such as the disclosure of Yahoo's account information of about 1 billion users and the disclosure of about 500 million pieces of user information sold on the dark web under Huazhu Hotel Group, which has caused great harm to users' privacy and corporate reputation [2]. To accelerate the construction of a secure and compliant data application system, countries have introduced data protection-related regulations one after another.

The rapid development of cloud computing, the Internet of things, the Internet of Vehicles, and other new-generation information technology makes the world enter the era of a data economy, all kinds of data grow rapidly and widely distributed, and artificial intelligence algorithms give full play to the value contained therein, prompting all occupations to digital and intelligent [3]. However, in the process of analyzing and processing data, a series of data security issues have been exposed. First, once the data are released to the public, it is uncontrolled, and it is difficult to prevent some attackers from inferring and exploiting the private information in the data. Second, the amount of data available to a single user is limited, and it is difficult to collaborate with multiple users to protect their data privacy with limited computing and communication resources [4]. In addition, users in open networks lack trust in each other, and trusted third parties usually face single point of failure attacks, lacking a decentralized mechanism for secure data sharing.

With the rapid development of IoT technology and the increasing popularity of 5G mobile communication services, IoT has become a megatrend of the fourth industrial revolution and human society has gradually started to step into the era of the interconnectedness of everything [5]. IoT is a very powerful distributed network, which consists of different connected entities (e.g., smart devices) that analyze and transmit the collected data and other operations, thus providing users with a comfortable living experience and making more appropriate decisions for development. The exchange of information and better integration of different types of application devices on the same task have led to the integration of the IoT access architecture from a vertical to a horizontal structure, resulting in a more complex process of obtaining data between devices [6]. The cross-domain collaboration of more heterogeneous devices has led to many small devices with weak computing power having to upload and outsource their computing tasks to specialized data processing centers through communication networks, with consequent issues such as the integrity of the outsourced data and the correctness of the outsourced results. Therefore, this study designs a multimedia fusion privacy protection model based on IoT data security to improve the security of existing IoT data processing [7]. In this model, the IoT data redundancy elimination method and differential privacy protection algorithm are successively used to remove duplicate data to obtain better quality data, and then privacy protection is applied to the IoT data to realize the secure processing of multimedia fusion data based on IoT data security. The rapid development of technology not only brings convenience to people but also generates serious privacy issues. If privacy issues are not taken seriously and solved, it will seriously restrict the innovation and development of technology.

## 2. Related Works

Professor Kevin Ashton of the Massachusetts Institute of Technology (MIT) first proposed the concept of IoT (Internet of things), that is, an intelligent service system that connects things, people, systems, and information resources through sensing devices according to agreed protocols to process and respond to information in the physical and virtual world, where “things” is a physical entity [8]. The Internet of things (IoT) has given rise to new application areas such as smart cities. The rapid development of the Internet of things has given birth to new application areas such as smart homes, smart cities, and personal smart wear. Through automatic sensing, data collection, intelligent control, and the Internet of things promote the creation of smart cities, in the field of transportation management, traffic control, and video surveillance to achieve fine management and optimization of services, improve urban efficiency, and benefit people's lives [9]. Currently, many cities in China are promoting “smart city” planning, mainly in public safety, transportation, medical, community, environmental protection, underground pipe network monitoring, water, education, and other fields. These applications are based on automatic sensing, data collection as a means, and intelligent control as the core, to achieve a comprehensive and integrated application of Internet of things technology. While the IoT industry is developing at a high speed, the application of new technologies also brings some security risks to people's lives [10]. If the IoT is attacked, it may lead to network paralysis, and those devices based on national security, personal health, and life and property safety may suffer serious threats and losses. Ahmed et al. constructed a password-based user authentication and data transmission scheme using a bilinear pairing algorithm. They combined user smart cards with passwords to avoid the insecurity of overly simple passwords that can be cracked [11]. Ciafrè et al. improved their scheme by proposing a two-factor authentication scheme for the Internet of things to achieve message reliability and be able to resist various types of security attacks [12]. At present, privacy issues have become one of the huge obstacles hindering the further development of network technology. If privacy protection cannot be guaranteed, all kinds of user data and related privacy may be stolen, which will seriously hinder individuals, enterprises, etc. trust, which in turn hinders the advancement of technology. Therefore, it is of great significance to study the privacy protection algorithm of multimedia fusion based on IoT data security under network regulations for privacy protection in the era of big data.

Privacy-preserving data release refers to the processing of sensitive information before the data are released to the public, which can maintain the privacy of the data and enable the released data to be used by analysts for value mining. The existing privacy-protected data release methods are mainly divided into two types: interactive and noninteractive [13]. Interactive methods, also known as online query methods, are designed by the data owner to meet the privacy-preserving nature of the interface for analysts to query the data and are commonly used by government agencies and R&D departments for external data release [14]. However, the usability of the query results obtained in this way is low, and too many queries will lead to large data errors; the noninteractive approach refers to the data owner cleaning the sensitive information in the original data before releasing it to the public, and the analysts will query the released data, which is commonly used in the release of various competition data sets. The focus of this approach is to protect user privacy while preserving the usability of the original data. Ghosn and Moses propose a differential privacy technique to distort sensitive attributes in the original data by adding noise while using a privacy budget to keep the data somewhat usable, where the privacy budget is used to control the degree of privacy protection achieved by the differential privacy technique, and the smaller the privacy budget, the higher the degree of privacy protection [15]. Riffaud et al. apply the differential privacy technique to a decision tree algorithm The differential privacy property is ensured by first fully generalizing the data, then subdividing the attributes layer by layer using an exponential mechanism, and finally adding Laplace noise to the published dataset [16]. However, this method wastes a large amount of privacy budget and adds excessive noise, resulting in the low usability of the released data. For this reason, Crosbie et al. improve the index mechanism scheme selection method to reduce the consumption of the privacy budget and improve the accuracy of published data [17].

In summary, some of the current studies on authentication schemes have proposed different approaches to ensure the security of the scheme, improve the efficiency of the scheme, protect user privacy, etc. However, some schemes cannot satisfy the system security while considering the efficiency of the scheme, and vice versa. Therefore, in this study, we propose a nonlinkable user anonymity scheme and a nonrepudiation privacy protection scheme, which not only protects user privacy but also solves various security problems and improves the operational efficiency of the system. Information leakage will allow users' various private information to be traded on the black market. It is easy to be disturbed by harassing calls and text messages such as promotion. If private information is sold to the hands of fraudulent gangs, it will cause the loss of user assets or even bankruptcy.

## 3. Multimedia Fusion Privacy Protection Algorithm Based on IoT Data Security

### 3.1. IoT Data Security Transmission Design

**Figure 1:**
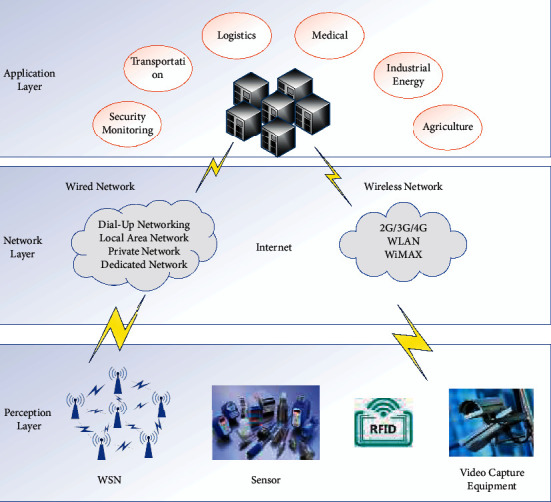
General application model diagram of the internet of things.

Nowadays, IoT is widely used in all occupations, and its typical application areas are mainly smart medical, smart industry, smart transportation, etc., which are gradually changing people's lives. The IoT network is composed of several parts, of which end user and sensing device are the two necessary parts. Among them, sensing devices are mainly used to collect information related to the surrounding environment, and end users can access the data collected by the sensing devices through the Internet [18]. In addition to the above two entities, there are usually some base stations (e.g., gateway nodes) in the IoT network to help the communication between end users and sensing devices. In addition, there will also be trusted third parties present in the IoT network whose role is to help in the registration of end users or sensing devices, generation of secret credentials, etc. The general application model of the IoT network is shown in Figure 1. With the continuous increase of the amount of data and the deepening of data mining technology, how to protect the privacy of users from being leaked while enjoying the service is an urgent problem to be solved. Privacy issues cover almost every corner of the Internet and have gradually become a huge stumbling block hindering the progress of Internet technology.

The booming digital economy is driving all industries toward digitization, networking, and intelligence, accompanied by various controversies over data security and personal privacy protection issues. Privacy computing technology provides a key technical path for data security compliance in convergence applications and has become a focus of attention for all sectors, including politics, academia, research, and industry. In the White Paper on Privacy Computing published by the Privacy Computing Consortium and the Cloud Institute of the China Academy of Information and Communications Technology in 2021, privacy computing is defined as a series of information technologies that analyze and calculate data on the premise of ensuring that the data provider does not disclose the original data, to ensure that the data are “available but not visible” in the process of circulation and integration.

In the digital society, everyone has a stronger demand for data production elements, both user services, and business marketing needs to use a large amount of data, especially in the distributed collaboration business model; all parties hope that the data can flow smoothly and reasonably reflect the value of data. However, contrary to this, data silos still exist and the rough use of data still needs to be solved. At the same time, legal compliance has become a major trend. Both domestically and internationally, laws and regulations related to personal information protection and data security have been introduced one by one, all of which put forward more stringent requirements for personal information protection and data security. This means that the security of data should be ensured and the privacy rights of individuals should be respected; in the whole life cycle of data, it is required to achieve comprehensive regulation and reach a compliant circulation.

**Figure 2:**
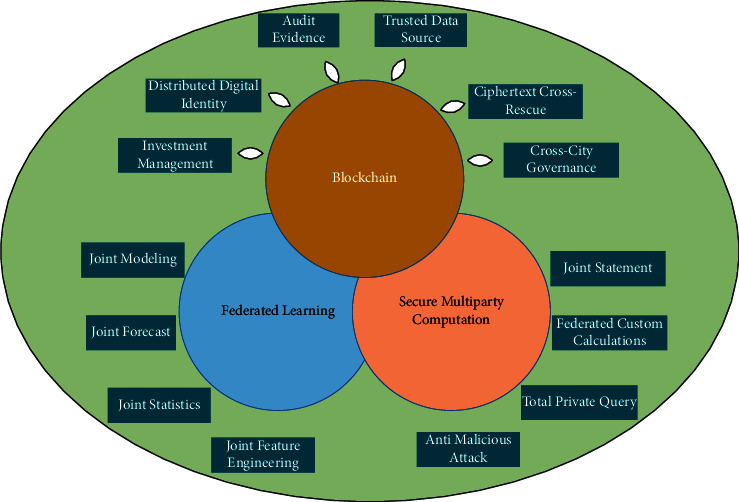
The three key core technologies of privacy computing relationship diagram.

On the one hand, in the real world, due to the scattered nature of data, low replication cost, and value aggregation, data are still highly scattered, and “data islands” are obvious; on the other hand, data with huge value can be replicated and used indefinitely at very low cost. The temptation of “gold mining” has led to various cases of data leakage, theft, and misuse [19]. To overcome these two challenges, privacy computing has emerged. The trend of building a digital society is to exchange data under the premise of security and privacy and provide high-quality and compliant services, which requires more innovations in technology, business model, and governance system. Initiatives such as the introduction of privacy computing in distributed systems and the development of compliant data exchanges reflect this spirit of innovation. In the field of privacy computing, blockchain, federated learning, and secure multiparty computing have become three key core technologies, and there are many overlaps and connections between these three technologies with mutual focus. From the perspective of blockchain, we can see that, on the one hand, data on the blockchain need to be protected by privacy algorithms; on the other hand, blockchain can also become the base and hub of collaboration in privacy computing; blockchain technology is used to record and trace the data sets, algorithm models, and computation processes in multiparty collaboration and evaluate and agree on the results to continuously optimize the efficiency of collaboration. The tripartite relationship between blockchain, federated learning, and secure multiparty computing is shown in Figure 2.

How differential privacy connects to other privacy computations is explained in the following steps:

1. Differential privacy is a technology in privacy computing, which is at the same level as algorithms such as homomorphic encryption, data desensitization, and obfuscation circuits. Each technology has a different focus. As mentioned earlier, differential privacy is more concerned with results, and there is no protection for the process of privacy computing.
2. Differential privacy can be used in secure multiparty computing and federated learning. They are equivalent to a subset of privacy computing. Differential privacy is an element of this subset, or a subset of the subset.
3. Secure multiparty computation is more like a protocol to me that can be used in federated learning. Federated learning is more of a framework for private computing, and other technologies, including TEE, are available. In the privacy computing architecture, we feel that algorithm applications basically describe federated learning.

Federated learning is an important framework for solving the problem of “data silos” by jointly utilizing data from multiple parties without taking user data out of the local context, and its core idea is that “data does not move the model moves, data is available but not visible.” Traditional centralized machine learning algorithms need to centralize data to a central server to train the model, which not only consumes a lot of communication resources but also faces the risk of privacy leakage during the data transmission. Secure multiparty computation (MPC) originated from the millionaire's problem proposed by Turing Award winner Yao Qizhi and refers to the technique of computing the agreed function without participants revealing their real data and relying on trusted third parties. Suppose 3 mutually untrusted distributed users *P*_1_, *P*_2_, and *P*_3_ agree to compute the function *y*_1_, *y*_2_, *y*_3_=*f*(*x*_1_, *x*_2_, *x*_3_), where *x*_*i*_(*i*=1,2,3) is the secret data held by the user *P*_*i*_ and *y*_*i*_ is the output obtained by user *P*_*i*_. Any user *P*_*i*_ has no access to any input information from other users except *y*_*i*_. Essentially, a blockchain is a distributed ledger that records transactions between parties in a transparent and tamper-proof manner. In addition to its application in the field of data currency, blockchain technology has been explored in several fields in recent years, such as edge computing, the Internet of things, and telematics, to establish a safe and secure transaction mechanism.

To ensure that the files uploaded to RSMR are not stolen, an asymmetric encryption algorithm ECC (elliptic curve cryptography) is used to encrypt the user's EMR in the encryption layer in this study. In this scheme, the user is given three digital passwords to be saved: a unique ID encrypted by SHA-256, public-private key pairs PK (public key), and SK (security key) encrypted by the ECC algorithm, and three digital passwords encrypted by IPNS (interplanetary name system) protocol after hashing. The user's unique ID is used to uniquely identify the user after being encrypted by the hash algorithm SHA-128, the public-private key pair PK encrypted by the ECC algorithm is used to encrypt the user's EMR, and a unique file ID (HashID) is obtained after encryption.

(1) $$\begin{array}{c} \text{SHA}+128\left(\rho \kappa ,\text{EMR}\right)=\text{HashID}. \end{array}$$

The user himself holds a public-private key pair (PK and SK) and gives the public key PK to the platform after the platform registration is completed. The platform uses formula (1) to encrypt the EMR of the user's medical record file to get the encrypted file Security File, and the encrypted record file is stored under the platform record folder address File Address for uploading to IPFS service.

To solve the problem of insufficient supervision of IoT devices and secure storage of IoT data, this study designs a blockchain-based smart contract model for Internet of things (BC-SC). The BC-SC model is different from the traditional IoT centralized architecture but uses blockchain as a trusted environment to work with IoT devices in a distributed architecture. For the traditional collection module, most of the data are collected through embedded computers and other devices, while in the blockchain IoT platform, the embedded computer devices will be assigned the corresponding access rights according to the smart contract rules to become the trusted nodes in the blockchain network and complete the collection of IoT data. The IoT platform can build the relevant blockchain platform and install the relevant blockchain modules on the operating system, and the platform also needs to provide rich interfaces for sensors and other control modules to complete the capture of signals. . The storage method of distributed ledger is used to create a secure data environment for user information and IoT data information, which ensures that the data are not tampered with or leaked. The nodes and user accounts in this system are based on cryptography technology, which uses public and private keys as the unique identifiers of devices and user accounts in the nodes and uses smart contract technology to reduce the human cost incurred in contract fulfillment, adjudication, and enforcement.

### 3.2. Multimedia Fusion Privacy Protection Algorithm Implementation

The raw data will be stored securely into the index chain using data indexes, and the specific content has each DP to keep locally. Meanwhile, to ensure the effectiveness of decentralized sharing, DR first needs to search for data of interest through SISC. DR and DP need to communicate with each other to request sharing authorization. After that, DR uses the shared coins to play DPS. The shared blocks are added to the vehicle blockchain with the help of proof of workload. The subjective logic mechanism is an evaluation criterion that uses the subjective logic of nodes to develop both sides of the interaction. The framework is based on the probabilistic fusion of subjective beliefs in the world and is specifically measured by subjective positive representations, negative representations, and uncertain representations. In addition, the subjective logic mechanism provides a rich set of logical operators to combine to associate different relational patterns:

(2) $$\begin{array}{c} L_{j}^{i}\approx \left\{\begin{array}{ccc} u_{j}^{i} & 0 & \Delta \\ 1 & \upsilon_{j}^{i} & 1\\ -1 & 0 & \delta_{j}^{i} \end{array}\right\}. \end{array}$$

Since reputation quantification from different perspectives gives different reputation values, what the model expects is slicing based on a high-quality reputation assessment. When reputation slicing is performed, the results from slicing are more reliable and realistic when the evaluator has more a priori knowledge of the data provider. In addition, reputation slicing is more effective when there is a contextual similarity between the results obtained by the evaluator and the respondent. Based on this, this model uses reputation slicing based on familiarity and timeliness. To ensure the security of data, it is also necessary to respect the privacy rights and interests of individuals; in the entire life cycle of data, it is required to achieve comprehensive specifications and achieve compliant circulation:

(3) $$\begin{array}{c} Acc_{x}=\sum_{i=0}\prod_{\gamma }^{n}g{\left(i\right)}^{a^{x}}. \end{array}$$

Distribution optimization is the optimization of the probability function of differential privacy, regardless of the data set. In these techniques of distribution optimization, differential privacy is usually achieved by adding Laplace noise or random noise computed by an exponential mechanism. Sensitivity calibration is the adjustment of the sensitivity to differential privacy. In these techniques of sensitivity calibration, the availability of data can be improved by calibrating the sensitivity to the optimal state. Both distribution optimization and sensitivity calibration perturb the data according to specific guidelines. However, when processing user data locally, sensitivity calibration does not apply to processing local data because there is no concept of sensitivity. In these techniques, a summary of the dataset is created by applying various decomposition, transformation, or compression techniques. The noise addition in these techniques is done to reduce the error rate and improve data availability while satisfying differential privacy. However, the communication cost of these techniques is high:

(4) $$\begin{array}{c} e\left(Acc_{x},g\right)=e^{\omega it_{x^{i}}}+e^{g\left(x^{i}\right)},i\geq 0. \end{array}$$

**Figure 3:**
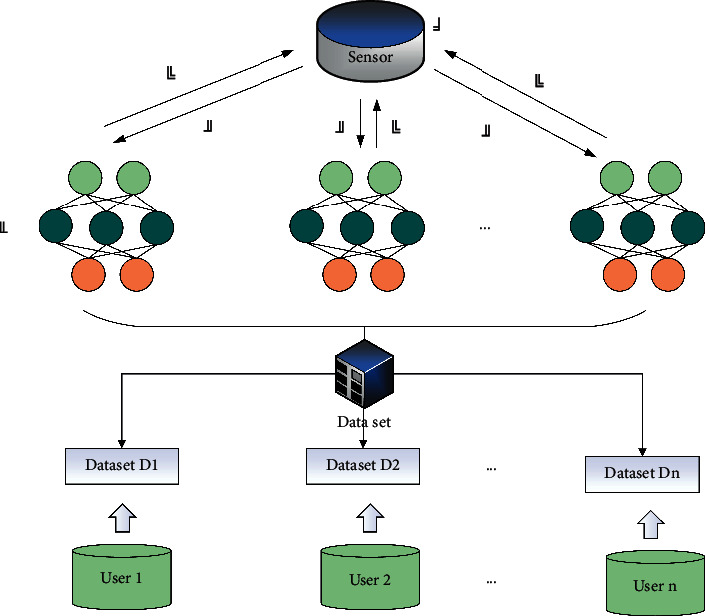
Local differential privacy framework.

While devices in different domains can be easily connected through the widely used network infrastructure, the secure communication that wants to be established between them is a daunting task because there are still many unresolved issues regarding trust and security. Domains do not necessarily trust each other because one domain is usually reluctant to give other domains access to its sensitive data, as shown in Figure 3. Most existing authentication mechanisms are built on PKI, where a trusted third party called certificate authority (CA) exists to provide certificates for authentication to all PKI. However, certificate management is costly, and the CA is vulnerable to potential attacks and prone to operational errors.

The arbitration mechanism designed in this model has the features of randomly setting the order of decision-makers in the decision process, invalidating the decision results without following the data, and resetting the decision-makers and decision order after one decision cycle, which can make the untrustworthy nodes unable to illegally obtain the data information in the arbitration process by applying for arbitration. On the premise of minimizing the network operation cost as well as the probability of data exposure, the network operation efficiency and the rights and interests of all parties are maximized:

(5) $$\begin{array}{c} {\text{Publickey}}_{\text{all}}={\sum_{i=1}{\text{ID}}_{\text{MD}}\left\|kr_{i}\right\|}^{p_{2}}. \end{array}$$

The logic of a smart contract acts like a rule that defines how transactions will be executed, how data will be processed, and the state of the distributed ledger. The smart contract can specify the functions of user interaction with the blockchain. The application running on the smart contract will receive transactions from the client and call the relevant functions to perform different types of operations on the distributed ledger, such as data processing and querying, storage of operational records for the execution of registration information, and some other operational transactions about the system [20]. The whole system will complete user registration and login, associate IoT devices and users, and give commands to IoT devices under the execution of smart contracts.

**Figure 4:**
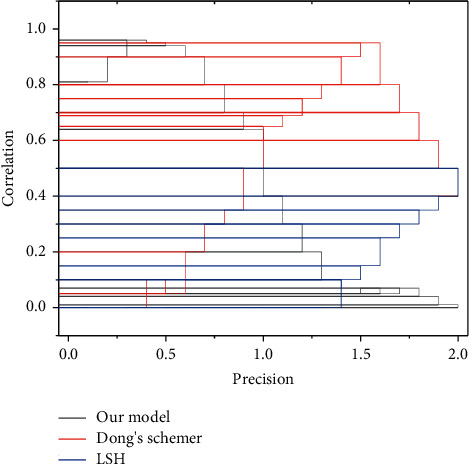
Effect of correlation on accuracy.

In this model, blockchain ensures that the shared records cannot be tampered with and ensures data traceability, while the automatic execution of smart contracts circumvents human intervention in data sharing, thus further protecting the secure sharing of data. For data providers, the decentralized architecture of this model based on the blockchain can protect against data security risks brought by data center storage and ensure that data are fully under their control. In addition, through this model, data providers can maximize the value of their data and get paid for it without worrying about privacy leakage and encourage other data owners to provide data, thus effectively solving the problem of “information silos.” Data with huge value can be copied and used infinitely at a very low cost. The temptation of this “savage nugget” has led to the occurrence of various data leakage, misappropriation, abuse, and other problem cases. During data sharing, the openness and transparency of the blockchain not only ensure the data privacy of the providers but also protect the intellectual property rights of data demanders to a certain extent, as shown in Figure 4. The anonymous operation of using pseudonyms during data storage and sharing also protects the identity of all parties from being leaked.

Based on the properties of differential privacy, the generator output is ensured to satisfy differential privacy by adding noise to the gradient of the discriminator in each training round. To speed up the convergence of the algorithm, an adaptive cropping threshold selection strategy is used to select a near-optimal cropping threshold for each parameter, and the weight parameters are clustered based on this value, and each class of parameters is gradient cropped with the same threshold and noise is added to reduce the consumption of privacy budget and improve the quality of the generated data. In addition, this study also proposes a dynamic privacy budget allocation strategy to effectively improve the stability of model training by allowing the noise to gradually decay during the training process. The privacy loss of the algorithm is accurately analyzed using moment statistics, and experiments are conducted on three real data sets. The results show that the data synthesized by this algorithm strictly satisfy differential privacy and have high usability for a variety of data analysis tasks. All interactive and transmitted data in SPN must reach distributed consistency through consensus among network nodes. Since the designed consensus algorithm is based on PBFT, the calculation time complexity analysis of service providers needs to consider the phase of PBFT.

The security of PDEC in this approach is built on a semihonest and complicit security model, i.e., the edge server and all edge devices will strictly follow the protocol steps for training, but at the same time, they may also presume the private data of other devices. Some devices may even conspire with the server or other devices to steal the private information of a target device, as listed in Table 1. This method assumes that an attacker can eavesdrop on messages transmitted during training, but cannot interrupt the message transmission process or inject toxic data, and does not consider the case of maliciously tampering with computation results or modifying local datasets to interfere with normal training.

The time consumed by arbitration in this model depends on the volume of data and the size of the model, and the change in the size of the data volume has a much greater impact on the efficiency of arbitration than the size of the model. Although arbitration consumes some time, the arbitration mechanism provides a legal guarantee for data sharing and can provide a fair, transparent, and efficient means to deal with the data privacy of both sharing parties.

## 4. Analysis of Results

### 4.1. Analysis of IoT Data Security Transmission Performance Results

In this model, multiple SPs are formed into a federated chain through a peer-to-peer network, and this network, i.e., the SPN, is set to interact with the meters. Since SPN has the decentralized property of blockchain, all the data interacted and transmitted in SPN must reach distributed consistency through consensus among network nodes [21]. Since the designed consensus algorithm is based on PBFT, the analysis of computational time complexity of service providers needs to examine the phase time consumption of PBFT, which is analyzed in the following. The MRE is positively correlated with the number of attributes. The more the number of attributes, the increase of the MRE, which reflects the accumulation process of the data aggregation server's estimation error of the original classification data frequency due to the increase of the data dimension.

**Figure 5:**
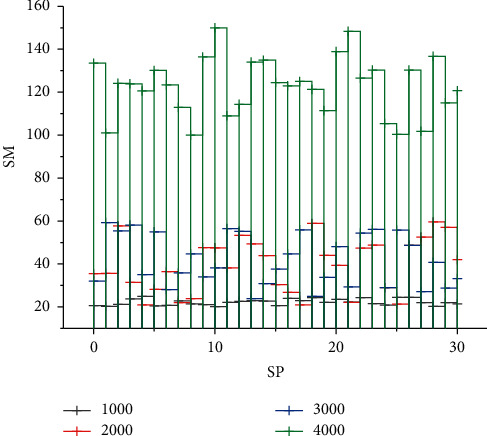
Time cost curve of expert node calculation based on different numbers of meters.

In this model, the computation time cost of each SP should include the master node election time, the communication time between the master node and the SM, and the slave node verification block time. Since none of the above three steps can be performed without the participation of the master node, the block validation operation performed by the slave node is to verify the transaction, which is only a part of the total SP computation time, and the time cost is much smaller, so only the computation time cost of the master node needs to be analyzed. When the meter initiates the session, i.e., before the transaction occurs, the expert node has been selected and the identity of the expert node does not change until that transaction completes the upstream operation in the block. The data computation rate of each slave node is the same, as shown in Figure 5.

**Figure 6:**
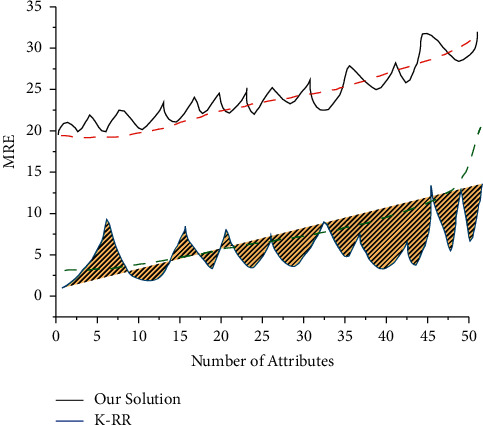
Effect of the number of attributes on the MRE.

From the figure, it can be observed that the slope of the curve shows a steep increase when the number of nodes in the SPN reaches 22 and then gradually decreases again as the number of nodes continues to increase. This is because the speed of message processing has a much greater impact on the time spent on consensus than the impact of the expert node selection time. Further analysis based on the above results shows that in SPN, the time cost of SP depends on the number of meters and the number of network nodes, i.e., the values of Num SM and Num SP. This is because when the number of nodes exceeds 21, the system automatically executes Algorithm 4.3 to select 21 transaction nodes from all the nodes in the current network. The time complexity of Algorithm 4.3 increases as the number of Num SM and Num SP increases, but the impact on the system is small. In addition, an increase in Num SM has a greater impact on Algorithm 4.3 than an increase in Num SP. However, even when Num SM is set to 10000, the computation time consumption of SPs still does not seriously affect the message transmission efficiency, as shown in Figure 6.

The MRE is positively correlated with the number of attributes, and the higher the number of attributes, the higher the MRE will be, which reflects the cumulative process of increasing the data dimensionality on the frequency estimation error of the original subtype data by the data aggregation server. Horizontally, our proposed method has less impact on MRE as the number of attributes increases, which is better than K-RR. Since the impact of message processing speed on consensus time is much greater than the impact of master node selection time, the slope of the curve will increase sharply and then gradually decrease as the number of nodes continues to increase.

Vertically, the MRE decreases as the user data increases, because the MRE is related to what is essentially the user's apportionment of the privacy budget, and as the number of users increases, the more data samples the data aggregation server can obtain. In both K-RR and our method, the data aggregation server can perform an unbiased estimation of the macroscopic statistics on the original data, so the more the data are, the more accurate the macroscopic quantity estimation is. On the other hand, our method is still superior to K-RR.

To ensure the validity of the model can be recognized by the industry, this experiment chooses Ether as the main experimental platform and conducts a comprehensive evaluation of the system performance through the test on that platform. Ethernet is one of the most popular blockchain application development platforms, which not only provides the traditional blockchain technical architecture but also implements smart contracts based on it, which greatly improves the development efficiency through automatic execution. From the above analysis of the experimental results, the proposed scheme is effective; in addition, this model uses blockchain to implement decentralized key management and provides a distributed consistency consensus mechanism to ensure the reliability and consistency of the system. In the analysis of the storage cost and time cost, the efficiency of the present model is optimal among the many existing schemes, but it can provide the most secure service while ensuring good efficiency compared to other schemes.

### 4.2. Analysis of Privacy Protection Algorithm Results

**Figure 7:**
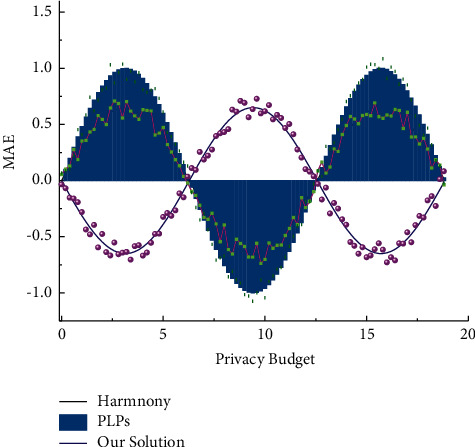
Impact of privacy budget on MAE.

To investigate the effect of the privacy budget *ε* on usability [1–15], and the effect of different privacy budgets on MAE, put the number of data attributes d = 20/10 and the number of users n = 10000/100, and the experimental results are shown in Figure 7.

Overall, the MAE decreases as the privacy budget increases. This is because the privacy budget essentially represents the degree of privacy protection by the user, and a larger privacy budget means that the user wants to protect privacy to a lesser extent, so the more accurate the user data obtained by the data collection platform is, and naturally, the more accurate the data collection platform estimates the original data, and thus the maximum absolute error will be correspondingly smaller. That is, if Σ⟶*∞*, then *MAE*⟶0. On the other hand, it is clear from Figure 7 that the method in this study outperforms both harmony and PLPS for different privacy budgets, and the data collection platform is more accurate in estimating the mean value of the original data when it has the perturbed data. When the data collection platform gets the perturbed data, the estimation of the statistics of the original data can be more accurate by using the method in this study.

The MAE is positively correlated with the number of attributes, i.e., an increase in the number of attributes leads to an increase in the maximum absolute error value, which essentially reflects the accumulation process of the error in the estimated value of the overall value of the original data by the data collection platform because of the increase in the dimensionality of the data. In the earliest literature, this accumulation of error with increasing dimensionality was equal to or even higher than linear.

**Figure 8:**
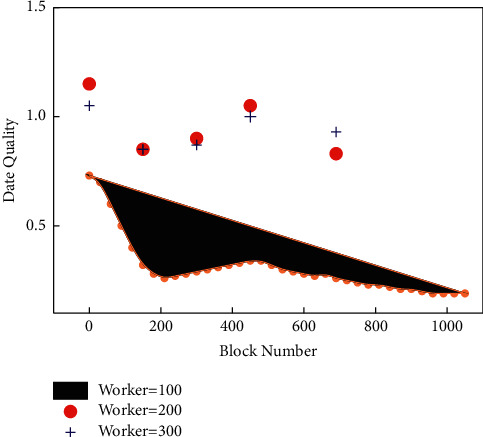
Privacy data quality relationship.

The blockchain is essentially a decentralized distributed database, and the blockchain itself is a series of data blocks with transaction information. Each data block is encrypted by a cryptographic mechanism and is connected in a certain chronological order. Each node in the blockchain network has an accounting function, and the transaction data are processed by cryptographic technology so that it cannot be tampered with and cannot be forged. From Figure 8, we can see that under the same block height, without considering the length of the task cycle, the impact of a different number of workers on data quality has a positive relationship, and the data quality becomes higher and higher as the number of workers increases. This is because, in the early stage of block generation, the evaluation parameters of tasks and the final reward allocation parameters of workers need to be adjusted continuously and optimally to achieve the best fairness; while after 200 block heights, the adjustment of evaluation parameters of the system decreases, and therefore the impact on data quality gradually decreases. In addition, it can be found that the data quality shows more fluctuations as the number of workers increases, which is because the increase in the number of workers brings a consequent increase in the computational load on the miners and therefore affects the task cycle of the workers and thus the data quality is determined based on the task cycle time. Although the above fluctuations are affected by the task cycle, such fluctuations have a negligible impact on the system during the entire operation of the mechanism.

From the figure, it can be concluded that an increase in the target value leads to an increase in the data quality assessment time, but the trend of increasing time slowly diminishes because although the data quality assessment is influenced by the target value, the increase in the target value triggers the dynamic adjustment of the system parameters to meet the block authentication requirements set by the system, and therefore this change gradually decreases as the system parameters are optimized. The blockchain can ensure that shared records cannot be tampered with and ensure data traceability, while the automatic execution of smart contracts avoids human intervention in data sharing, thereby further protecting the safe sharing of data.

This section uses federated learning in privacy computing techniques as a tool to address the problem of efficient privacy data collaboration. First, for the edge computing scenario, a sparse bidirectional compression algorithm is designed to reduce the communication overhead, and a privacy protection protocol based on secret sharing and homomorphic encryption is proposed to resist the conspiracy attack among edge devices. Second, for the cloud computing scenario, an efficient training strategy is designed based on the characteristics of federated learning to reduce the number of interactions between cloud servers and users, and a lightweight privacy protection protocol is designed to apply to this strategy with less computational overhead compared to similar methods. Security analysis and experimental results show that the two efficient privacy data collaboration methods proposed in this section can not only guarantee data privacy theoretically but also perform better in terms of communication overhead, computation overhead, data availability, and resistance to privacy attacks and complicity attacks, respectively.

## 5. Conclusion

In this study, during the process of designing the IoT data security processing model based on edge computing, the IoT data redundancy elimination method based on information entropy suppression and differential privacy protection algorithm are used to obtain better quality data after the privacy protection of IoT data, which has a positive effect on the application effect of this study's model and has certain usability. When the model in this study deals with the security of IoT data based on edge computing when the privacy protection budget value is 0.6, the information leakage ratio is significantly lower than that at 0.4; when the decision tree depth value is 6, the information leakage ratio is the smallest and the information security is the most significant; after the use of the model in this study, the information leakage ratio is significantly lower and the use effect is better. For cloud computing scenarios with a high latency such as interest recommendation and joint marketing, efficient federation training strategy is proposed to speed up the training efficiency of the model, and a lightweight privacy protection protocol is designed to apply the strategy, which protects the privacy of the data with a small computational overhead. The security analysis and experimental results show that the two efficient privacy data collaboration methods based on federated learning proposed in this study can effectively improve privacy in the multiparty collaborative modeling process with less computational overhead, communication overhead, and higher data availability.

## Figures and Tables

**Table 1:** Effect of data volume and model size on arbitration time.

Model size (MB)		10	20	50	100
The amount of data (MB)	50	11.3	5.5	11.5	11.9
	100	22	19.4	15.1	19.1
	200	27.6	24.1	23.9	33
	500	61.8	61.3	76.1	77.3

## Data Availability

The datasets used and/or analyzed during the current study are available from the corresponding author upon reasonable request.
